# Feasibility of the Medial Temporal lobe Atrophy index (MTAi) and derived methods for measuring atrophy of the medial temporal lobe

**DOI:** 10.3389/fnagi.2014.00305

**Published:** 2014-11-05

**Authors:** Francisco Conejo Bayón, Jesús Maese, Aníbal Fernandez Oliveira, Tamara Mesas, Estibaliz Herrera de la Llave, Tania Álvarez Avellón, Manuel Menéndez-González

**Affiliations:** ^1^Fundación de NeurocienciasOviedo, Spain; ^2^Grupo de Trabajo Reumatología Basada en la Evidencia, Sociedad Española de ReumatologíaMadrid, Spain; ^3^Psychology, Universidad de OviedoOviedo, Spain; ^4^Hospital Álvarez-BuyllaMieres, Spain; ^5^Morphology and Cellular Biology, Universidad de OviedoOviedo, Spain; ^6^Instituto de NeurocienciasOviedo, Spain

**Keywords:** feasibility, reproducibility, MTAi, yrMTA, 2D-MTA, yrRMTA, planimetry

## Abstract

**Introduction**: The Medial Temporal-lobe Atrophy index (MTAi), 2D-Medial Temporal Atrophy (2D-MTA), yearly rate of MTA (yrRMTA) and yearly rate of relative MTA (yrRMTA) are simple protocols for measuring the relative extent of atrophy in the medial temporal lobe (MTL) in relation to the global brain atrophy. Albeit preliminary studies showed interest of these methods in the diagnosis of Alzheimer’s disease (AD), frontotemporal lobe degeneration (FTLD) and correlation with cognitive impairment in Parkinson’s disease (PD), formal feasibility and validity studies remained pending. As a first step, we aimed to assess the feasibility. Mainly, we aimed to assess the reproducibility of measuring the areas needed to compute these indices. We also aimed to assess the efforts needed to start using these methods correctly.

**Methods**: A series of 290 1.5T-MRI studies from 230 subjects ranging 65–85 years old who had been studied for cognitive impairment were used in this study. Six inexperienced tracers (IT) plus one experienced tracer (ET) traced the three areas needed to compute the indices. Finally, tracers underwent a short survey on their experience learning to compute the MTAi and experience of usage, including items relative to training time needed to understand and apply the MTAi, time to perform a study after training and overall satisfaction.

**Results**: Learning to trace the areas needed to compute the MTAi and derived methods is quick and easy. Results indicate very good intrarater Intraclass Correlation Coefficient (ICC) for the MTAi, good intrarater ICC for the 2D-MTA, yrMTA and yrRMTA and also good interrater ICC for the MTAi, 2D-MTA, yrMTA and yrRMTA.

**Conclusion**: Our data support that MTAi and derived methods (2D-MTA, yrMTA and yrRTMA) have good to very good intrarater and interrater reproducibility and may be easily implemented in clinical practice even if new users have no experience tracing the area of regions of interest.

## Introduction

The recent focus on biomarkers in the diagnosis of Alzheimer’s disease (AD) have created a need to translate research findings into tools for use in everyday clinical practice. Although AD and mild cognitive impairment (MCI) are commonly diagnosed using criteria based in clinical findings, MRI findings may aid the clinical diagnosis, and may predict clinical progression. New research criteria have recently been proposed for AD, and MCI that incorporate (disproportionate) medial temporal lobe (MTL) or hippocampal atrophy on MRI as one of the supportive features.

Age-associated differences are detected in the MTL with an acceleration of Medial Temporal Lobe Atrophy (MTA) starting around 72 years of age in healthy people (Jack et al., 2004; Salk et al., 2014). However, these changes are modest and their rate of progression over time is relatively slow with a mean rate of about 1.6% per year. Accelerated MTA is a consistent finding in AD and MCI with rates of about 2.8% in stable MCI, 3.7% in MCI transitioning to AD (MCI progressors), and up to 4.0% in AD. Frontotemporal dementia may also lead to MTA, but in a different pattern: behavioral frontotemporal dementia and semantic dementia show atrophy in the anterior portion of the hippocampus, and in semantic dementia the atrophy is asymmetrical, with the left hippocampus being affected more severely. No significant hippocampal atrophy is detected in non-fluent progressive aphasia (Mesulam et al., 2014). Other diseases such as dementia with Lewy bodies do not show MTA or it is much milder (Menéndez-González et al., 2014).

All these changes can be measured on brain MRI using different approaches on structural MRI. Volumetric techniques quantify the volume of regions of interest on *ad-hoc* MRI studies that are quantified by using well manual well automatic image analysis. Planimetry methods are conceived to be used on standard MRI studies in routine clinical practice by measuring the area of regions of interest manually. Linear methods use measures of the width or height of brain structures, including the ventricular system or spaces around the brain cortex. Visual assessment rating scales are a quick way of assessing atrophy of the MTL on one single coronal MRI slice straightforward, and can be performed easily in the clinical setting; the disadvantage is that these scales are totally subjective and there is a loss of accuracy compared with objective analysis (Scheltens et al., 1992).

In contrast to MTA, ventricular enlargement in old people lacks specificity, representing a measure of global brain atrophy and is strongly associated with aging both in healthy and diseased people. In addition, almost any neurodegenerative disorder affecting the brain hemispheres leads to some degree of ventricular enlargement, and so do some psychiatric conditions. Thus, it is interesting to compare measures indicative of atrophy in the MTL with measures indicative of global brain atrophy. This can be done using volumetry (3D) or planimetry (2D).

Shortly, the “Medial Temporal-lobe Atrophy index” (MTAi), is a simple method for measuring the relative extent of atrophy in the MTL in relation to the global brain atrophy (Menéndez-González, 2014b). This 2D-method consists on calculating a ratio using the area of three regions traced manually on one single coronal MRI slide. High values are suggestive of atrophy in the MTL out of proportion to other brain structures, and therefore the pattern of atrophy matches the expected in typical AD (Galton et al., 2001; van de Pol et al., 2006).

Albeit preliminary studies showed interest of planimetric methods for diagnosing AD, frontotemporal lobe degeneration (FTLD) and cognitive impairment in Parkinson’s disease (PD)^1^, formal feasibility and validity studies remained pending. As a first step, we aimed to assess the feasibility of the MTAi and derived methods: 2D-Medial Temporal Atrophy (2D-MTA), yearly rate of MTA (yrRMTA) and tyearly rate of relative MTA (yrRMTA). Mainly, we aimed to assess the reproducibility of measuring the areas needed to compute these indices. Reproducibility refers to the degree of agreement between measurements or observations conducted on replicate specimens by different people, as part of the precision of a test method. Test–retest variability can be caused by intra-individual variability and intra-observer variability. These parameters have paramount importance when validating a new diagnostic test (Bossuyt et al., 2003); and some recommendations on the validation of biomarkers for diagnosing dementias have also remarked the importance of assessing them in first term (McGhee et al., 2014; Noel-Storr et al., 2014). We also aimed to assess the efforts needed to start using these methods correctly.

## Methods

A series of 230 1.5T-MRI studies of subjects ranging 65–85 years old who had been studied for cognitive impairment were used in this study. Six inexperienced tracers (IT) plus one experienced tracer (ET) took part in this study. IT had different backgrounds in life sciences, basic neuroanatomical knowledge, and no previous experience in tracing areas of brain regions at all. The ET is one of the researchers who described the methods and has extensive experience using them.

**Figure 1:**
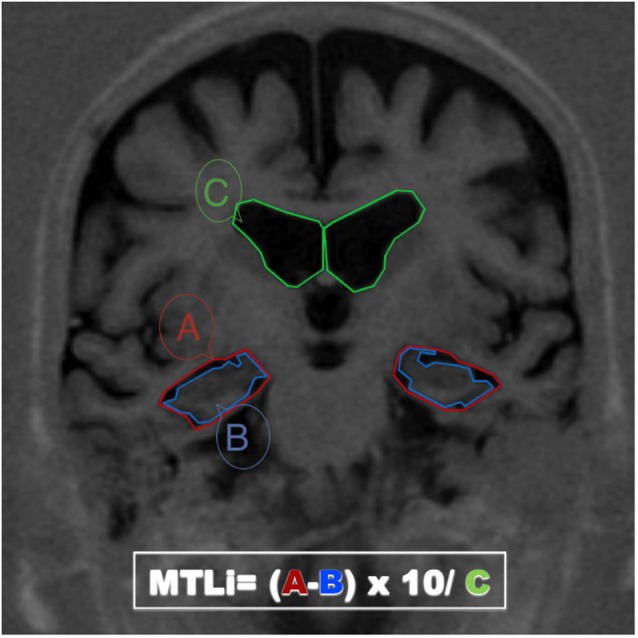
**MRI coronal section passing through the interpeduncular fosae**. The boundaries of the three areas needed for calculating the Medial Temporal Atrophy index (MTAi) and derived methods have been drawn in three different colors: (1) the medial temporal lobe region (A, red), defined in a coronal brain slide as the space bordered in its inferior side by the tentorium cerebelli, in its medial side by the cerebral peduncles, in its upper side by the roof of the temporal horn of the lateral ventricle and in its lateral side by the colateral sulcus and a straight-line linking the colateral sulcus with the lateral edge of the temporal horn of the lateral ventricle; (2) the parenchima within the medial temporal region, that includes the hippocampus and the parahippocampal girus (B, blue); and (3) the body of the ipsilateral lateral ventricle (C, green).

First, the IT read the protocol of the MTAi (Menéndez-González, 2014a). They were in charge of installing the DICOM software, loading MRI studies, selecting the appropriate slide and tracing the three areas needed to compute the MTAi on each hemisphere (right and left A, B and C) according to the original protocol, on a number of MRI studies ranging from 20 to 120 cases. The IT used different DICOM viewers depending on the operating system installed in their computers: 3 IOS users used Osirix™ and 3 Windows users used ONIS™. The areas traced by the IT were corrected by the ET in all cases. The MTAi consists on calculating a ratio using the area of three regions traced manually on one single coronal MRI slide at the level of the interpeduncular fossa: (1) the MTL region (A), defined in a coronal brain slide as the four-sided space bordered in its inferior side by the tentorium cerebelli, in its medial side by the cerebral peduncles, in its upper side by the roof of the temporal horn of the lateral ventricle and in its lateral side by the collateral sulcus and a straight-line linking the collateral sulcus with the lateral edge of the temporal horn of the lateral ventricle; (2) the parenchima within the medial temporal region, that includes the hippocampus and the parahippocampal gyrus—the fimbria taenia and plexus choroideus are excluded—(B); and (3) the body of the ipsilateral lateral ventricle (C) (Figure 1). Therefrom we can compute the “2D-Medial Temporal Atrophy” *(2D-MTA = A−B)* that represents absolute atrophy of the MTL; and the ratio “Medial Temporal Atrophy index” * (MTAi = (A−B) × 10/C)* that represents the relative atrophy of the MTL in comparison with the enlargement of the lateral ventricles, that represent global brain atrophy. The MTAi is suitable to assess the asymmetry of relative MTA within a subject. High asymmetry is typical of some types of FTLD. However, as there is important inter-individual variability in the size of the lateral ventricles, this index is not recommended for comparing subjects but to track the progression in a given subject over time. Indeed, if we have 2 MRI studies from different times (1 = first one, 2 = second one), we can also compute the yrMTA as follows: *yrMTA = (A2−B2)−(A1−B1) × 1200/(#months between MRI studies)* and the yearly rate of relative MTA (yrRMTA) as follows: *yrRMTA=(A2−B2)−(A1−B1) × 1200/(C2−C1) × (# months between MRI studies)*.

Finally, tracers underwent a short survey on their experience learning to compute the MTAi and experience of usage, including items relative to training time needed to understand and apply the MTAi (number of attempts needed until the ET verified the tracing was correct), time needed to perform a study after training (timed by the own tracers, in minutes) and overall satisfaction (measured using a simple qualitative scale: easy/normal/hard).

Statistical analyses were performed with the softwares R™ and Epidat™. Computation of the MTA, 2D-MTA, yrMTA and yrRMTA were made following the formulas explained above (Menéndez-González et al., 2014). The reproducibility was assessed using the Intraclass Correlation Coefficient (ICC)-Model 2, one by one for every of the three areas needed to compute the MTAi on each hemisphere and for the final MTAi results (right and left). We used a qualitative scale to qualify the strength of concordance as very good (>0.90), good (0.80–0.90), moderate (0.60–0.80) and poor (<0.60).

## Results

### Reproducibility: results from the test-retest studies

In total, IT traced areas from 290 MRI studies from 230 cases, 60 of which also had a 1-year follow-up MRI study. Ninety studies were traced twice by the same IT (180 sets of results), and 200 were traced twice by 2 different IT (400 sets of results). We used the set of 180 double-traced by same-tracer results to assess the intrarater reproducibility and the set of 400 double-traced by different-tracer results to assess the interrater reproducibility. Thirty cases of the each set corresponded to the 1 year follow-up MRI. Thus, we computed the yrMTA and the yrRMTA in 60 cases and the MTAi and 2D-MTA in 230 cases. Results of intrarater and interrater reproducibility are shown in Tables 1, 2 respectively.

**Table 1:** **Intrarater Intraclass Correlation Coefficient and strength of concordance among the different areas needed to compute the MTAi, 2D-MTA, yrMTA and yrRMTA**.

	CCI	CI 95%		Strength
	Mean	Inferior	Superior
rA	0.87	0.68	0.91	Good
lA	0.86	0.66	0.90	Good
rB	0.83	0.71	0.89	Good
lB	0.84	0.76	0.87	Good
rC	0.95	0.92	0.94	Very good
lC	0.94	0.91	0.96	Very good
rMTAi	0.91	0.90	0.92	Very good
lMTAi	0.92	0.91	0.93	Very good
r2D-MTA	0.88	0.80	0.95	Good
l2D-MTA	0.85	0.81	0.89	Good
ryrMTA	0.82	0.76	0.89	Good
lyrMTA	0.84	0.77	0.90	Good
ryrRMTA	0.81	0.77	0.87	Good
lyrRMTA	0.83	0.75	0.89	Good

*r: right hemisphere; l: left hemisphere*.

**Table 2:** **Interrater Intraclass Correlation Coefficient and strength of concordance among the different areas needed to compute the MTAi, 2D-MTA, yrMTA and yrRMTA**.

	CCI	CI 95%		Strength
	Mean	Inferior	Superior
rA	0.87	0.69	0.92	Good
lA	0.86	0.72	0.91	Good
rB	0.83	0.69	0.89	Good
lB	0.84	0.71	0.89	Good
rC	0.88	0.73	0.91	Very good
lC	0.90	0.87	0.92	Very good
rMTAi	0.88	0.82	0.92	Good
lMTAi	0.87	0.83	0.91	Good
r2D-MTA	0.84	0.79	0.90	Good
l2D-MTA	0.85	0.81	0.91	Good
ryrMTA	0.83	0.74	0.89	Good
lyrMTA	0.83	0.75	0.90	Good
ryrRMTA	0.82	0.75	0.88	Good
lyrRMTA	0.81	0.76	0.86	Good

*r: right hemisphere; l: left hemisphere*.

### Satisfaction: results from the survey

IT needed to train with 2–5 cases (mean 3 cases) before being able to compute the MTAi on their own correctly. After training, IT needed between 4 and 7 min (mean 5 min) to examine a new case. All tracers rated the method as “easy to learn” and “easy to apply”.

## Discussion

One of the strengths of planimetry methods is that can be measured using almost any of the DICOM softwares commonly used by clinicians or radiologists to visualize medical images worldwide. Most of these softwares are intuitive and require little or no training at all. Learning to trace the areas needed to compute the MTAi and derived methods is quick and easy even for naive tracers. Even more importantly, tracing these areas have good intra- and interrater reproducibility. As expected, the area with the best intra- and interrater ICC was area C—the lateral ventricle—since it has the easiest anatomical limits. Areas A and B had poorer intrarater and interrater ICC since anatomical limits are somewhat more complicated. However, the intrarater and interrater ICC for areas A and B is still good enough to yield very good intrarater ICC for the MTAi, good intrarater ICC for the 2D-MTA, yrMTA and yrRMTA and also good interrater ICC for the MTAi, 2D-MTA, yrMTA and yrRMTA. These results are comparable to those from automatic volumetry (Hsu et al., 2002; Wolz et al., 2014) and much better than those from manual volumetry and visual scales (Scheltens et al., 1997; Hsu et al., 2002). Particularly, assessment of cerebral atrophy using visual rating scales is totally subjective and has moderate intrarater and poor interrater reproducibility (Scheltens et al., 1997).

Accurate manual volumetric assessment requires standard operating procedures that include the know-how specific for the modality, acquisition parameters, and extensive learning and *ad-hoc* softwares that also require training to be used correctly (Frisoni et al., 2014). In addition, harmonization of manually segmented hippocampus is still in progress (Frisoni and Jack, 2011). Thus, manual volumetric methods have a steep learning curve while the MTAi while derived 2D methods have a learning curve with a quick start. The MTAi and derived methods may be easily implemented for estimating MTA in clinical practice, even if new users have no experience tracing the area of regions of interest. Indeed, any health professional with basic neuroanatomical knowledge can take these measures after short training. The MTAi and derived methods can also readily be incorporated into a standardized radiological report and may also be useful in clinical trials.

## Conclusions

In conclusion, results from this feasibility study support that the MTAi and derived methods (2D-MTA, yrMTA and yrRMTA) have good to very good intrarater and interrater reproducibility and may be easily implemented for estimating MTA in clinical practice, even if new users have no experience tracing the area of regions of interest.

## Conflict of interest statement

The authors declare that the research was conducted in the absence of any commercial or financial relationships that could be construed as a potential conflict of interest.

## Footnotes

^1^http://brainatrophyindices.blogspot.com
